# Ambroxol effects in glucocerebrosidase and α‐synuclein transgenic mice

**DOI:** 10.1002/ana.24790

**Published:** 2016-11-18

**Authors:** Anna Migdalska‐Richards, Liam Daly, Erwan Bezard, Anthony H. V. Schapira

**Affiliations:** ^1^ Department of Clinical Neurosciences, Institute of NeurologyUniversity College LondonLondonUnited Kingdom; ^2^ Neurodegenerative Diseases InstituteUniversity of BordeauxMixed Unit of Research 5293BordeauxFrance; ^3^ Neurodegenerative Diseases Institute, National Center for Scientific ResearchMixed Unit of Research 5293BordeauxFrance

## Abstract

**Objective:**

Gaucher disease is caused by mutations in the glucocerebrosidase 1 gene that result in deficiency of the lysosomal enzyme glucocerebrosidase. Both homozygous and heterozygous glucocerebrosidase 1 mutations confer an increased risk for developing Parkinson disease. Current estimates indicate that 10 to 25% of Parkinson patients carry glucocerebrosidase 1 mutations. Ambroxol is a small molecule chaperone that has been shown to increase glucocerebrosidase activity in vitro. This study investigated the effect of ambroxol treatment on glucocerebrosidase activity and on α‐synuclein and phosphorylated α‐synuclein protein levels in mice.

**Methods:**

Mice were treated with ambroxol for 12 days. After the treatment, glucocerebrosidase activity was measured in the mouse brain lysates. The brain lysates were also analyzed for α‐synuclein and phosphorylated α‐synuclein protein levels.

**Results:**

Ambroxol treatment resulted in increased brain glucocerebrosidase activity in (1) wild‐type mice, (2) transgenic mice expressing the heterozygous L444P mutation in the murine glucocerebrosidase 1 gene, and (3) transgenic mice overexpressing human α‐synuclein. Furthermore, in the mice overexpressing human α‐synuclein, ambroxol treatment decreased both α‐synuclein and phosphorylated α‐synuclein protein levels.

**Interpretation:**

Our work supports the proposition that ambroxol should be further investigated as a potential novel disease‐modifying therapy for treatment of Parkinson disease and neuronopathic Gaucher disease to increase glucocerebrosidase activity and decrease α‐synuclein and phosphorylated α‐synuclein protein levels. Ann Neurol 2016;80:766–775

Homozygous mutations in the glucocerebrosidase 1 (*GBA1*) gene have long been known to cause Gaucher disease (GD), the most common lysosomal storage disorder. More recently, it has been observed that both GD patients and carriers have an increased risk of developing Parkinson disease (PD). Both homozygous and heterozygous *GBA1* mutations cause a reduction of glucocerebrosidase (GCase) activity and confer a 20‐ to 30‐fold increased risk for PD.^1^, ^2^, ^3^, ^4^ It is estimated that approximately 10 to 25% of PD patients have a *GBA1* mutation (PD‐*GBA1*), with the most common mutations being L444P and N370S, and the highest frequency in Ashkenazi patients.^1^, ^2^, ^3^, ^4^

The clinical presentation of PD‐*GBA1* is very similar to idiopathic PD, except for a slightly younger age of onset and a tendency to more cognitive impairment. Importantly, the pathology of PD‐*GBA1* is identical to idiopathic PD, with nigral dopamine cell loss, Lewy bodies, and neurites containing α‐synuclein, so although the exact mechanism by which *GBA1* mutations increase the risk for PD is still unknown, it is likely that as in idiopathic PD, accumulation of α‐synuclein, especially α‐synuclein phosphorylated at serine 129 (S129), plays an important role in the development and progression of PD‐*GBA1*.^5^, ^6^

Several studies have highlighted the reciprocal relationship between GCase activity and α‐synuclein. It has been shown in SH‐SY5Y cell cultures, neuronal cultures, conduritol‐β‐epoxide (CβE)‐treated mice, and transgenic *Gba1* mouse models that reduced GCase activity results in increased α‐synuclein levels.^7^, ^8^, ^9^, ^10^, ^11^, ^12^, ^13^, ^14^ Conversely, it has been demonstrated in cell models that increased α‐synuclein causes a decrease in GCase activity.^15^ Moreover, the biochemical analysis of *GBA1* wild‐type Parkinson patients showed that GCase activity and protein levels were significantly reduced in several brain regions,^15^, ^16^ further stressing the importance of GCase in PD development.

The increasing evidence linking GCase with α‐synuclein in both PD‐*GBA1* and idiopathic PD patients suggests that treatments capable of increasing GCase might be beneficial to PD patients both with and without *GBA1* mutations. To this end, small molecular chaperones designed to cross the blood–brain barrier that are capable of increasing GCase activity are being investigated as a novel therapy for PD to decrease α‐synuclein levels.^17^, ^18^, ^19^, ^20^, ^21^, ^22^, ^23^, ^24^, ^25^

One such small molecular chaperone is ambroxol hydrochloride (ambroxol). Ambroxol was identified as a GCase chaperone after screening the library of US Food and Drug Administration–approved drugs with a thermal denaturation assay using wild‐type GCase.^19^ To date, 2 ambroxol studies using wild‐type mice or transgenic mice carrying a human transgene containing either N370S or L444P mutation failed to provide convincing evidence to determine whether ambroxol is capable of increasing GCase in the peripheral and neuronal tissues.^23^, ^24^ Considering the potential importance of ambroxol as a novel treatment for PD and neuronopathic forms of GD, we have investigated the effect of ambroxol on wild‐type mice, on mice expressing the L444P mutation in the murine *Gba1* gene, and on mice overexpressing human α‐synuclein in the absence of mouse α‐synuclein.

## Materials and Methods

### Materials

4‐Methylumbelliferyl β‐D‐glucopyranoside, 4‐methylumbelliferyl N‐acetyl‐β‐D‐glucosaminide, sodium taurocholate hydrate, and ambroxol hydrochloride were purchased from Sigma‐Aldrich (St Louis, MO). Pierce BCA Protein Assay, Halt Protease Inhibitor Cocktail, Halt Phosphatase Inhibitor, Pierce ECL Western Blotting Substrate, and Power SYBR Green PCR Master Mix were purchased from Thermo Scientific (Waltham, MA). Luminata Forte Western HPR Substrate was purchased from Millipore (Billerica, MA). RNeasy Mini Kit was purchased from Qiagen (Hilden, Germany). Precision nanoScript 2 Reverse Transcription kit (RT‐nano‐Script2) was purchased from Primerdesign (Chandler's Ford, UK). Anti–α‐synuclein antibody (4D6; ab1903), anti–α‐synuclein (phospho S129) antibody (EP1536Y; ab51253), anti–mitochondrial transcription factor A (TFAM) antibody (ab131607), and anti–transcription factor EB (TFEB) antibody ‐ ChIP grade (ab2636) were purchased from Abcam Biochemicals (Cambridge, UK). Polyclonal swine antirabbit immunoglobulins/horseradish peroxidase (HRP), polyclonal goat antimouse immunoglobulins/HRP, and polyclonal rabbit antigoat immunoglobulins/HRP were purchased from Dako (Glostrup, Denmark).

### Mice

Mice were treated in accordance with local ethical committee guidelines and the UK Animals (Scientific Procedures) Act of 1986. All procedures were carried out in accordance with Home Office guidelines (United Kingdom). B6129SF1/J (101043) mice expressing wild‐type *Gba1* (wild‐type mice) and FVB;129S6‐*Snca* ^*tm1Nbm*^ Tg(SNCA)1Nbm/J (010710) mice overexpressing human α‐synuclein in the absence of endogenous mouse α‐synuclein (*SNCA*/*SNCA* mice) were purchased from Jackson Laboratory (Bar Harbor, ME). B6;129S4‐Gbatm1Rlp/Mmnc (000117‐UNC) mice expressing heterozygous knockin L444P mutation in the murine *Gba1* gene (*L444P*/ + mice) were purchased from the Mutant Mouse Regional Resource Center.^26^ Transgenic mice containing heterozygous L444P mutation were identified by polymerase chain reaction (PCR) of ear genomic DNA using forward primer 5′‐TGTGAAGTTCCTGGATGCCTATG‐3′ and reverse primer 5′‐TGGTGATGTCTACAATGATGGGAC‐3′. Only male mice were used. All mice were 10 to 12 weeks of age at the start of treatments.

### Ambroxol Administration

Ambroxol was dissolved in distilled water by vigorous shaking. Ambroxol solution was given to mice instead of normal drinking water, and mice had access to it 24 hours per day. Ambroxol solution was changed daily. To establish the optimum concentration of ambroxol, wild‐type mice were split into 5 groups (5–6 mice per group) and given ambroxol at concentrations of 1, 3, 4, and 5mM respectively for 12 consecutive days. Untreated control mice were given distilled water that was changed daily for 12 consecutive days. At the end of treatment, 4 different regions of the brain (brainstem, midbrain, cortex, and striatum) were collected. After the optimal concentration of ambroxol was established (as explained in Results), *L444P*/ + and *SNCA*/*SNCA* mice were evaluated.

### Enzyme Assays

Brain samples were homogenized in 5mM ethylenediaminetetraacetic acid, 750mM sodium chloride, 50mM Tris (pH 7.4), 10% Triton X‐100, unless stated otherwise. Homogenate was centrifuged to remove insoluble materials, and protein concentration was determined using a Pierce BCA Protein Assay. Resulting lysate was diluted to 2mg/ml in distilled water and sonicated.

GCase activity was measured in lysate (20 μg protein) using 5mM 4‐methylumbelliferyl β‐D‐glucopyranoside substrate in McIlvaine buffer (pH 5.4) supplemented with 22mM sodium taurocholate hydrate at 37 °C for 1 hour. The reaction was stopped by adding 0.25M glycine (pH 10.4), and substrate fluorescence was measured at excitation of 365nm, emission of 450nm with a PerkinElmer (Waltham, MA) fluorescence spectrometer.^27^ All GCase assays were performed in duplicate. GCase activity was expressed as nanomoles of substrate released per milligram protein per hour.

β‐Hexosaminidase (HEXB) was measured in lysate (2 μg protein) using 2mM 4‐methylumbelliferyl N‐acetyl‐β‐D‐glucosaminide substrate in McIlvaine buffer (pH 4.2) at 37 °C for 30 minutes. The reaction was stopped by adding 0.25M glycine (pH 10.4), and substrate fluorescence was measured as above.^28^ All HEXB assays were performed in triplicate. HEXB activity was expressed as nanomoles of substrate released per milligram protein per minute.

### Total, Cytosolic, and Lysosomal Fractions

A subset of brain samples were used to obtain the total, cytosolic, and lysosomal fraction. These brain samples were homogenized in a lysis buffer containing 250mM sucrose, 10mM Tris (pH 7.4), and 1mM ethylenediaminetetraacetic acid (10 μl of buffer per 1mg of brain sample). About 20% of homogenate (total fraction) was collected into a separate tube. The remaining homogenate was centrifuged at 1,500 relative centrifugation force (rcf) for 10 minutes. The supernatant was collected into a fresh tube. The remaining pellet was further homogenized in the lysis buffer (80% of initial buffer's volume) and centrifuged at 1,500rcf for 10 minutes, and the supernatant was collected and combined with the previously collected supernatant. Combined supernatants were centrifuged at 1,500rcf for 10 minutes before the subsequent supernatant was collected into a fresh tube and centrifuged again at 17,000rcf for 20 minutes. The resulting supernatant corresponded to the cytosolic fraction. The remaining pellet was washed and then resuspended in the lysis buffer (20% of initial buffer's volume). The resulting lysate corresponded to the lysosomal fraction. GCase and HEXB activity of the total, cytosolic, and lysosomal fraction were measured as above.

### Western Blotting

Brain samples were homogenized in 10mM Tris (pH 7.4.), 0.1% sodium dodecyl sulfate, 1 × Halt Protease Inhibitor Cocktail, and 1 × Halt Phosphatase Inhibitor Cocktail. Homogenate was centrifuged to remove insoluble materials, and protein concentration was determined using a Pierce BCA Protein Assay. Supernatant (30 μg protein) was separated on 12% NuPAGE Tris‐Bis gels, transferred to Hybond‐P membrane, and probed with primary and respective secondary antibodies. Bands were detected by Pierce ECL Western Blotting Substrate or Luminata Forte Western HRP Substrate (Millipore), and band intensity was measured using the ChemiDoc MP System (Bio‐Rad, Hercules, CA). Protein expression was expressed as a ratio against β‐actin.

### Quantitative Real‐Time PCR

RNA was extracted from mouse brains using RNeasy kit. RNA was converted to cDNA using RT‐nano‐Script2, and relative mRNA levels were measured using Power SYBR Green PCR Master Mix. Relative expression of α‐synuclein and GCase mRNA was measured with Power SYBR Green PCR Master Mix using a STEP One PCR machine (Applied Biosystems, Foster City, CA). β‐actin mRNA levels were used to normalise data. Primers are listed in Table 1. Relative expression was calculated using the ΔC_T_ method.

**Table 1:** Primers for Quantitative Real‐Time Polymerase Chain Reaction

Target	Sequence	Annealing Temperature, °C
Mouse β‐actin	5′‐TACAGCTTCACCACCACAGC‐3′, 5′‐AAGGAAGGCTGGAAAAGAGGC‐3′	58
Mouse GCase	5′‐GACCAACGCTTGCTGCTAC‐3′, 5′‐ACAGCAATGCCATGAACGTA‐3′	58

GCase = glucocerebrosidase.

### Statistical Analysis

Data are expressed as mean ± standard error of the mean, and statistical significance between groups was analyzed with the unpaired *t* test or 1‐way analysis of variance (ANOVA), followed by the Tukey Honestly Significant Difference (HSD) test.

## Results

### Establishing the Optimum Concentration of Ambroxol in Wild‐Type Mice

**Figure 1:**
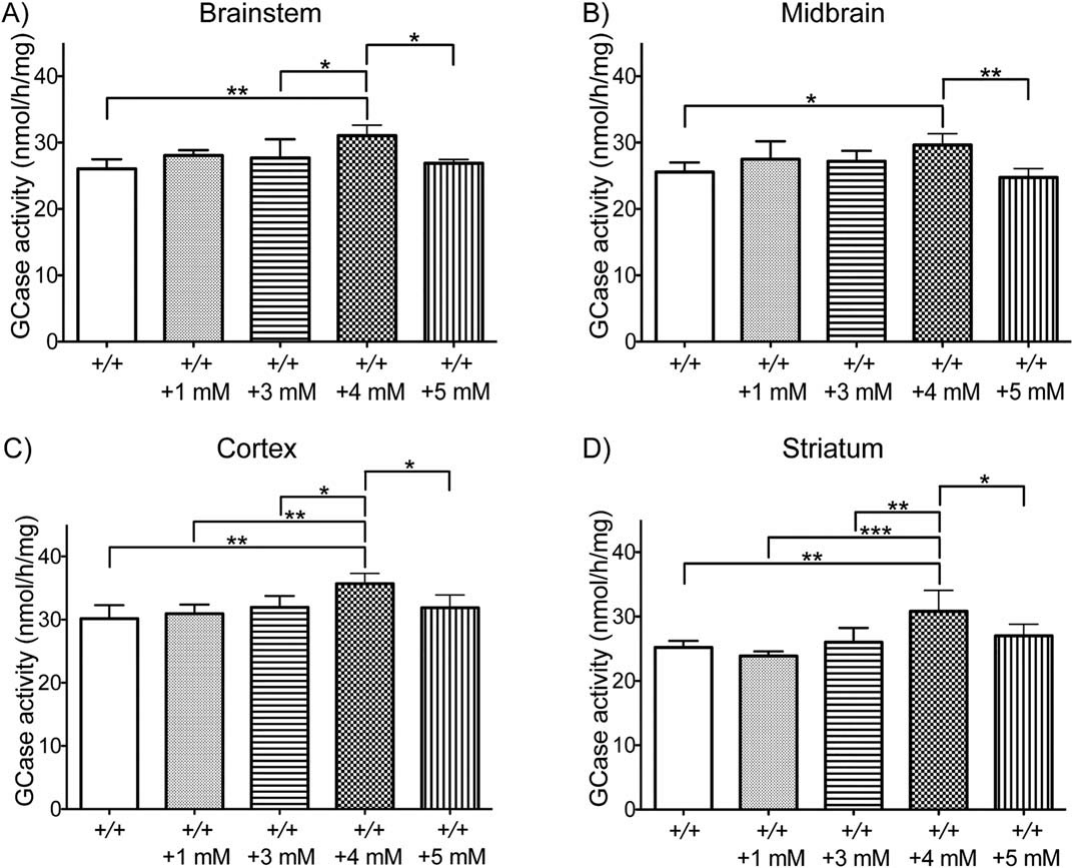
Glucocerebrosidase enzyme (GCase) activity in wild‐type mouse brains after 12 days of treatment with 1, 3, 4, or 5mM ambroxol. GCase activity was significantly increased in the brainstem, midbrain, cortex, and striatum of mice treated with 4mM ambroxol (n = 5), when compared to untreated controls (n = 6). Data were analyzed with the 1‐way analysis of variance test, followed by the post hoc analysis using the Tukey Honestly Significant Difference test. *p < 0.05, **p ≤ 0.01, ***p ≤ 0.001.

GCase activity was measured in the brainstem, midbrain, cortex, and striatum of wild‐type mice given 0, 1, 3, 4, or 5mM ambroxol for 12 consecutive days. The 1‐way ANOVA analysis showed a statistically significant difference in GCase activity between groups in the brainstem (*F*_4,22_ = 5.115, *p* = 0.0046), midbrain (*F*_4,21_ = 5.373, *p* = 0.0039), cortex (*F*_4,22_ = 5.849, *p* = 0.0023), and striatum (*F*_4,20_ = 7.711, *p* = 0.0006). The post hoc analysis using the Tukey HSD test determined that GCase activity was significantly increased in the brainstem (19%), midbrain (16%), cortex (18%), and striatum (22%) of mice treated with 4mM ambroxol (but not of mice treated with 1, 3, and 5mM ambroxol), when compared to untreated mice (Fig 1). The significant increase in GCase activity in mice treated with 4mM ambroxol was also observed in the cortex and striatum (when compared to mice treated with 1, 3, and 5mM ambroxol), in the brainstem (when compared to mice treated with 3 and 5mM ambroxol), and in the midbrain (when compared to mice treated with 5mM ambroxol; see Fig 1). The increase in GCase activity had no apparent effect on HEXB activity, another lysosomal enzyme in mice treated with 1 to 4mM ambroxol, but there was a significant decrease in HEXB activity observed in the brainstem (19%, *p* = 0.0035) and midbrain (13%, *p* = 0.0109) of mice treated with 5mM ambroxol (data not shown). Taking into consideration the above data, a 4mM concentration of ambroxol was chosen as the optimal dose, and was used in all subsequent experiments.

### Ambroxol Treatment Did Not Change GCase mRNA Levels in Wild‐Type Mice

The quantitative real‐time PCR was conducted in the brainstem, midbrain, cortex, and striatum of ambroxol‐treated and untreated wild‐type mice to determine whether the significant increase in GCase activity was a consequence of increased GCase mRNA levels. No significant changes in GCase mRNA levels were observed (Table 2).

**Table 2:** GCase mRNA levels, and TFAM and TFEB Protein Levels in Wild‐Type Mice Treated with 4mM Ambroxol

Measure	Brainstem	Midbrain	Cortex	Striatum
GCase mRNA levels	↑ 3%, *p* = 0.44	↑ 15%, *p* = 0.07	↑ 9%, *p* = 0.59	↑ 7%, *p* = 0.49
TFAM/β‐actin	↓ 11%, *p* = 0.42	—	↓ 11%, *p* = 0.60	—
TFEB/β‐actin	↓ 13%, *p* = 0.39	—	↑ 4%, *p* = 0.74	—

Five mice treated with 4mM ambroxol and 6 untreated mice were analyzed. Data were analyzed with the unpaired *t* test.

GCase = glucocerebrosidase.

### Ambroxol Treatment Did Not Affect TFAM and TFEB Protein Levels in Wild‐Type Mice

Ambroxol has been reported to upregulate the CLEAR (coordinated lysosomal expression and regulation) pathway by increased transcription of TFEB.^23^ Mitochondria function is modified by GCase deficiency,^12^ and so we investigated whether ambroxol had an effect on TFEB and TFAM levels. TFEB and TFAM protein levels were measured by Western blotting analysis in the brainstem and cortex of ambroxol‐treated and untreated wild‐type mice. No significant changes in protein levels of TFEB and TFAM were observed in the brainstem or cortex of wild‐type mice treated with 4mM ambroxol, when compared to untreated littermates (see Table 2).

### Ambroxol Treatment Increased GCase Activity in L444P/ + Mice

**Figure 2:**
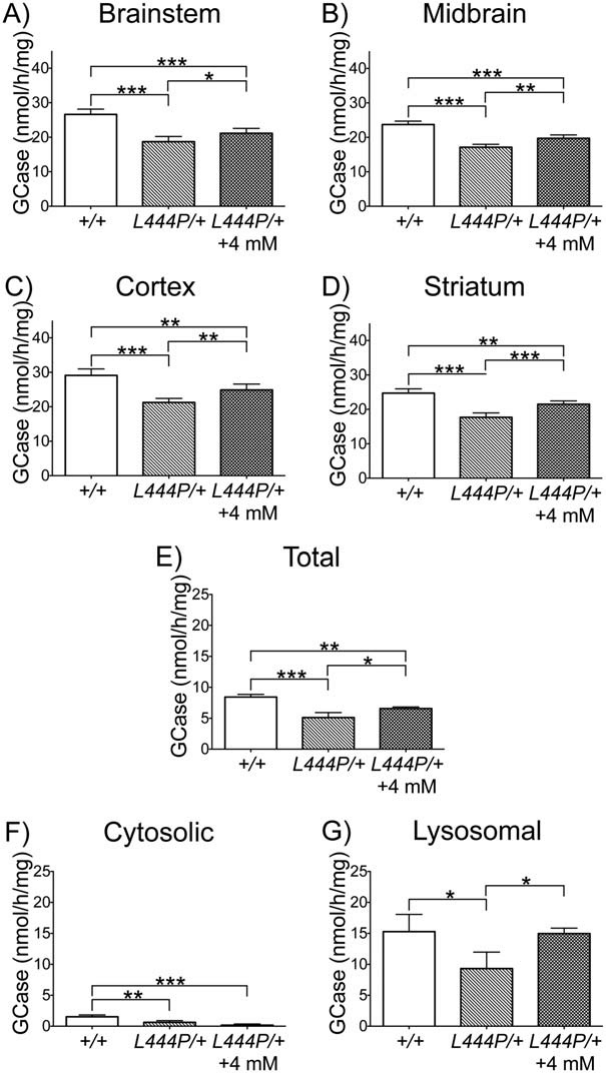
Glucocerebrosidase enzyme (GCase) activity in *L444P*/ + mouse brains after 12 days of treatment with 4mM ambroxol. (A–D) Baseline GCase activity was significantly decreased in the brainstem, midbrain, cortex, and striatum of untreated *L444P*/ + mice (n = 5), when compared to untreated +/ + littermates (n = 5). After 4mM ambroxol treatment, GCase activity was significantly increased in the brainstem, midbrain, cortex, and striatum of *L444P*/ + mice (n = 7), when compared to untreated *L444P*/ + littermates (n = 5). (E–G) Baseline GCase activity was significantly decreased in the total, cytosolic, and lysosomal fractions of the brainstem of untreated *L444P*/ + mice (n = 4), when compared to nontreated +/ + littermates (n = 4). After 4mM ambroxol treatment, GCase activity was significantly increased in the total and lysosomal, but not the cytosolic fraction of the brainstem of *L444P*/ + mice (n = 4), when compared to untreated *L444P*/ + littermates (n = 4). Data were analyzed with the 1‐way analysis of variance test, followed by the post hoc analysis using the Tukey Honestly Significant Difference test. *p < 0.05, **p ≤ 0.01, ***p ≤ 0.001.

To determine whether ambroxol was capable of increasing GCase activity in *Gba1* transgenic mice, GCase activity was measured in the brainstem, midbrain, cortex, and striatum of *L444P*/ + mice given 0 or 4mM ambroxol and +/ + littermates given 0mM ambroxol for 12 consecutive days. The 1‐way ANOVA analysis showed a statistically significant difference in GCase activity between groups in the brainstem (*F*_2,13_ = 37.92, *p* < 0.0001), midbrain (*F*_2,13_ = 56.02, *p* < 0.0001), cortex (*F*_2,15_ = 32.70, *p* < 0.0001), and striatum (*F*_2,12_ = 45.42, *p* < 0.0001). The post hoc analysis using the Tukey HSD test showed that baseline GCase activity was significantly decreased in the brainstem (30%), midbrain (28%), cortex (27%), and striatum (29%) of untreated *L444P*/ + mice, when compared to untreated +/ + littermates (Fig 2A–D). The Tukey HSD analysis also determined that GCase activity was significantly increased in the brainstem (13%), midbrain (15%), cortex (17%), and striatum (21%) of *L444P*/ + mice treated with 4mM ambroxol, when compared to untreated mice (see Fig 2A–D). HEXB activity in the brainstem, midbrain, cortex, and striatum was similar between untreated *L444P*/ + and +/ + mice (data not shown). Ambroxol treatment did not have an effect on HEXB in the brainstem, midbrain, cortex, and striatum of *L444P*/ + mice (data not shown).

GCase activity was also measured in the total, cytosolic, and lysosomal fractions of the brainstem of *L444P*/ + mice given 0 or 4mM ambroxol and untreated +/ + littermates. The 1‐way ANOVA analysis showed a statistically significant difference in GCase activity between groups in the total (*F*_2,7_ = 34.67, *p* = 0.0002), cytosolic (*F*_2,7_ = 24.35, *p* = 0.0007), and lysosomal fractions (*F*_2,7_ = 6.445, *p* = 0.0259). The post hoc analysis using the Tukey HSD test determined that GCase activity was significantly decreased in the total (39%), cytosolic (58%), and lysosomal (39%) fractions of the brainstem of untreated *L444P*/ + mice, when compared to untreated +/ + littermates (see Fig 2E–G). The Tukey HSD analysis also showed that GCase activity was significantly increased in the total (29%) and lysosomal (61%) fractions of the brainstem of *L444P*/ + mice treated with 5mM ambroxol, when compared to untreated ones (see Fig 2E–G). Interestingly, ambroxol treatment of *L444P*/ + mice led to by far the greatest increase of GCase activity in the lysosomal fraction (the only place where GCase is functional), with activity almost identical to GCase activity of untreated +/ + mice.

### GCase Activity Is Reduced in SNCA/SNCA Mice and Is Restored by Ambroxol

**Figure 3:**
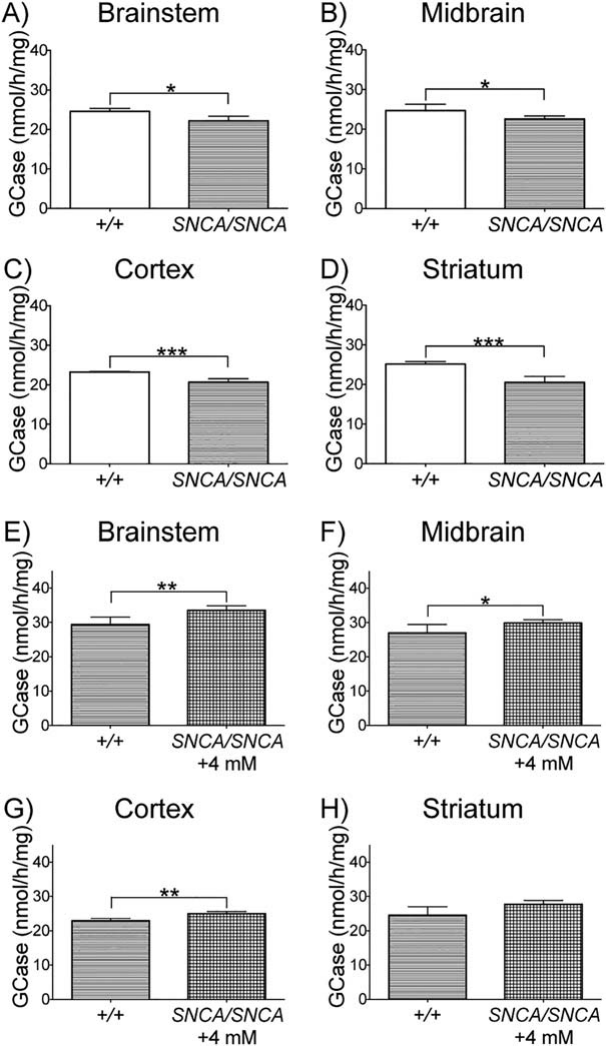
Glucocerebrosidase enzyme (GCase) activity in *SNCA/SNCA* mouse brains after 12 days of treatment with 4mM ambroxol. (A–D) Baseline GCase activity was significantly decreased in the brainstem, midbrain, cortex, and striatum of untreated *SNCA/SNCA* mice (n = 6), when compared to untreated +/ + mice (n = 4). (E–H) After 4mM ambroxol treatment, GCase activity was significantly increased in the brainstem, midbrain, and cortex, but not in the striatum of *SNCA/SNCA* mice (n = 6), when compared to untreated *SNCA/SNCA* littermates (n = 6). Data were analyzed with the unpaired *t* test. *p < 0.05, **p ≤ 0.01, ***p ≤ 0.001 versus control.

GCase activity was measured in the brainstem, midbrain, cortex, and striatum of *SNCA/SNCA* mice given 0 or 4mM ambroxol and wild‐type mice given 0mM ambroxol for 12 consecutive days. Baseline GCase activity was significantly decreased (as analyzed by the unpaired *t* test) in the brainstem (10%), midbrain (9%), cortex (11%), and striatum (18%) of untreated *SNCA/SNCA* mice, when compared to untreated wild‐type mice (Fig 3A–D). GCase activity was significantly increased (as analyzed by the unpaired *t* test) in the brainstem (14%), midbrain (11%), cortex (9%), and striatum (13%) of *SNCA/SNCA* mice treated with 4mM ambroxol, when compared to untreated littermates (see Fig 3E–H). HEXB activity in the brainstem, midbrain, cortex, and striatum was unchanged between untreated *SNCA/SNCA* and +/ + mice (data not shown). The increase in GCase activity in *SNCA/SNCA* mice after ambroxol treatment had no significant effect on HEXB activity in the brainstem, midbrain, cortex, and striatum (data not shown).

### Ambroxol Treatment Decreased α‐Synuclein and Phospho‐α‐Synuclein Protein Levels in SNCA/SNCA Mice

**Figure 4:**
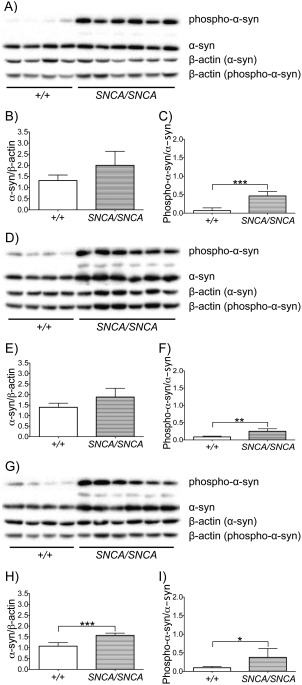
α‐Synuclein (α‐syn) and serine 129 (S129)‐phosphorylated α‐synuclein protein levels in *SNCA/SNCA* mouse brains. (A) Western blotting for α‐synuclein and S129‐phosphorylated α‐synuclein protein in the brainstem (example blots shown). (B) α‐Synuclein protein levels were increased in the brainstem of *SNCA/SNCA* mice (n = 6), when compared to +/ + mice (n = 4), but this increase did not reach statistical significance. (C) S129‐phosphorylated α‐synuclein protein levels were significantly increased in the brainstem of *SNCA/SNCA* mice (n = 6), when compared to +/ + mice (n = 4). (D) Western blotting for α‐synuclein and S129‐phosphorylated α‐synuclein protein in the cortex (example blots shown). (E) α‐Synuclein protein levels were increased in the cortex of *SNCA/SNCA* mice (n = 6), when compared to +/ + mice (n = 4), but this increase did not reach statistical significance. (F) S129‐phosphorylated α‐synuclein protein levels were significantly increased in the cortex of *SNCA/SNCA* mice (n = 6), when compared to +/ + mice (n = 4). (G) Western blotting for α‐synuclein and S129‐phosphorylated α‐synuclein protein in the striatum (example blots shown). (H) α‐Synuclein protein levels were significantly increased in the striatum of *SNCA/SNCA* mice (n = 6), when compared to +/ + mice (n = 4). (I) S129‐phosphorylated α‐synuclein protein levels were significantly increased in the striatum of *SNCA/SNCA* mice (n = 6), when compared to +/ + mice (n = 4). Data were analyzed with the unpaired *t* test. *p < 0.05, **p ≤ 0.01, ***p ≤ 0.001 versus control.

To check the level of α‐synuclein overexpression in *SNCA/SNCA* mice, Western blotting analysis was conducted to measure α‐synuclein protein levels in the brainstem, cortex, and striatum of untreated *SNCA/SNCA* and wild‐type mice. α‐Synuclein protein levels were significantly increased in the striatum (47%) of untreated *SNCA/SNCA* mice, when compared to untreated wild‐type mice (unpaired *t* test, *p* = 0.0003; Fig 4G, H). α‐Synuclein protein levels in the brainstem and cortex of untreated *SNCA/SNCA* mice were increased by 52% and 35%, respectively, when compared to untreated wild‐type mice, but these changes did not reach statistical significance (unpaired *t* test, *p* = 0.0764 and *p* = 0.0635, respectively; see Fig 4A, B, D, E). Next, to determine whether the increase in α‐synuclein protein levels was accompanied by an increase in the phosphorylation of α‐synuclein in *SNCA/SNCA* mice, Western blotting analysis was conducted to measure the levels of phosphorylation of α‐synuclein at S129 in the brainstem, cortex, and striatum of untreated *SNCA/SNCA* and wild‐type mice. Significant increases in S129 phosphorylation of α‐synuclein were observed in the brainstem (589%), cortex (189%), and striatum (285%) of untreated *SNCA/SNCA* mice, when compared to untreated wild‐type mice (unpaired *t* test, *p* = 0.0004, *p* = 0.0026, and *p* = 0.0493, respectively; see Fig 4A, C– E, G, I).

**Figure 5:**
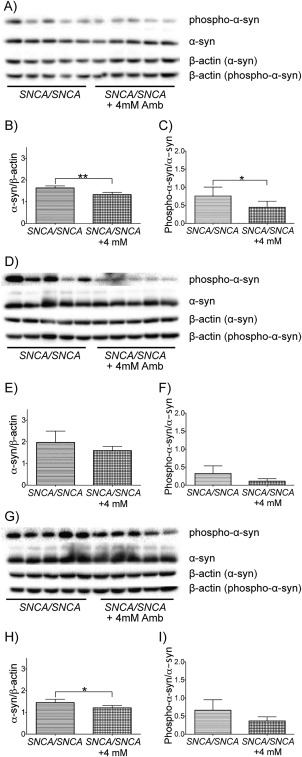
α‐Synuclein (α‐syn) and S129‐phosphorylated α‐synuclein protein levels in *SNCA/SNCA* mouse brains after 12 days of treatment with 4mM ambroxol (Amb). (A) Western blotting for α‐synuclein and serine 129 (S129)‐phosphorylated α‐synuclein protein in the brainstem (example blots shown). (B) α‐Synuclein protein levels were significantly decreased in the brainstem of ambroxol‐treated *SNCA/SNCA* mice (n = 5), when compared to untreated littermates (n = 5). (C) S129‐phosphorylated α‐synuclein protein levels were significantly decreased in the brainstem of ambroxol‐treated *SNCA/SNCA* mice (n = 5), when compared to untreated littermates (n = 5). (D) Western blotting for α‐synuclein and S129‐phosphorylated α‐synuclein protein in the cortex (example blots shown). (E) α‐Synuclein protein levels were decreased in the cortex of ambroxol‐treated *SNCA/SNCA* mice (n = 5), when compared to untreated littermates (n = 5), but this decrease did not reach statistical significance. (F) S129‐phosphorylated α‐synuclein protein levels were decreased in the cortex of ambroxol‐treated *SNCA/SNCA* mice (n = 5), when compared to untreated littermates (n = 5), but this decrease did not reach statistical significance. (G) Western blotting for α‐synuclein and S129‐phosphorylated α‐synuclein protein in the striatum (example blots shown). (H) α‐Synuclein protein levels were significantly decreased in the striatum of ambroxol‐treated *SNCA/SNCA* mice (n = 5), when compared to untreated littermates (n = 5). (I) S129‐phosphorylated α‐synuclein protein levels were decreased in the striatum of ambroxol‐treated *SNCA/SNCA* mice (n = 5), when compared to untreated littermates (n = 5), but this decrease did not reach statistical significance. Data were analyzed with the unpaired *t* test. *p < 0.05, **p ≤ 0.01 versus control.

To determine whether 4mM ambroxol treatment had an effect on protein expression of α‐synuclein and on S129 phosphorylation of α‐synuclein, protein levels were measured by Western blotting analysis in the brainstem, cortex, and striatum of ambroxol‐treated and untreated *SNCA/SNCA* mice. α‐Synuclein protein levels were significantly decreased in the brainstem (19%) and striatum (17%) of ambroxol‐treated *SNCA/SNCA* mice, when compared to untreated littermates (unpaired *t* test, *p* = 0.0012 and *p* = 0.0236, respectively; Fig 5). α‐Synuclein protein levels in the cortex of ambroxol‐treated *SNCA/SNCA* mice were decreased by 19%, when compared to untreated littermates, but this change did not reach statistical significance (unpaired *t* test, *p* = 0.1737). Significant decrease in S129 phosphorylation of α‐synuclein was observed in the brainstem (41%) of ambroxol‐treated *SNCA/SNCA* mice, when compared to untreated littermates (unpaired *t* test, *p* = 0.0490). Decreases in S129 phosphorylation of α‐synuclein were also observed in the cortex (65%) and striatum (45%) of ambroxol‐treated *SNCA/SNCA* mice, when compared to untreated littermates, but these changes did not reach statistical significance (unpaired *t* test, *p* = 0.0656 and *p* = 0.0681, respectively).

## Discussion

This study provides the first comprehensive analysis of ambroxol treatment on GCase activity in different brain regions in wild‐type, *L444P*/+, and human α‐synuclein overexpressing mice.

We observed a significant increase of GCase activity in the brainstem, midbrain, cortex, and striatum following 4mM ambroxol administration in distilled water. This further supports both the ability of ambroxol to cross the brain–blood barrier and its ability to enhance the activity of wild‐type GCase.^23^ This may reflect ambroxol's ability to chaperone wild‐type GCase trafficking from endoplasmic reticulum to lysosome, or through an effect on the CLEAR pathway.^23^, ^24^, ^25^ We did not observe any serious adverse effects of ambroxol administration in wild‐type mice. At higher concentrations ambroxol resulted in decrease in daily water intake, which may be related to high osmolality of ambroxol at high concentrations. The observed decline in water consumption probably explains why 4mM but not 5mM ambroxol resulted in significant increase in GCase activity, as mice given 5mM ambroxol probably did not receive as much ambroxol as the mice treated with 4mM ambroxol. The only other study that investigated ambroxol effect on GCase activity in wild‐type mice found a significant increase of activity in the cerebellum, but no increase in the cerebrum after 1 week of treatment.^24^ However, several differences between the study designs, such as dose optimization or treatment length and solvent used, may explain why we were able to observe the effect of ambroxol in different brain regions of our wild‐type mice. Moreover, it is likely that levels of GCase activity vary among different brain regions, and so a collective analysis of the cerebrum as a whole (rather than the individual regions we considered here) may also account for the difference in our results.

The increase in GCase activity was not concomitant with an increase in GCase mRNA levels in ambroxol‐treated mice. This suggests that the increase in GCase activity is likely to be regulated through other components of the lysosomal pathway, such as saponin C, which has been shown to both activate and stabilize GCase.^29^, ^30^

It has recently been suggested that in fibroblasts ambroxol might increase GCase activity by activating the genes of the CLEAR network through the action of TFEB, a key regulator of lysosomal biogenesis.^23^ Ambroxol treatment of control, Gaucher, and Parkinson‐*GBA1* fibroblasts led to a significant upregulation of TFEB mRNA. Our data showed no significant increase in TFEB protein levels in wild‐type mice following ambroxol treatment. This difference might simply relate to in vitro versus in vivo effects, dose, bioavailability, or alternatively to tissue or species specificity. We also evaluated whether ambroxol had an effect on TFAM, an important regulator of mitochondrial transcription, but did not observe any changes in TFAM protein levels.

The *L444P*/ + mice had a significant reduction in brain GCase activity levels. A significant increase of GCase activity was observed in the brainstem, midbrain, cortex, and striatum of *L444P*/ + mice following 4mM ambroxol administration in distilled water. The measurement of GCase activity in different fractions of the brainstem of *L444P*/ + mice treated with ambroxol showed that by far the greatest increase in GCase activity occurred in lysosomes, restoring levels comparable to GCase activity of untreated +/ + mice. To our knowledge, the only other ambroxol study conducted on transgenic mice with *GBA1* mutations used mice carrying a human transgene containing either the N370S or L444P mutation. These mice showed no significant increase of GCase activity in the cerebrum after a subcutaneous injection of ambroxol (100mg/kg for 14 days).^25^ This failure to increase GCase activity may reflect dose or limited availability of the drug within this route in contrast to our optimized protocol for ambroxol administration.

Finally, we investigated transgenic mice overexpressing human α‐synuclein in the absence of endogenous mouse α‐synuclein. First, we addressed the question of the potential reciprocal relationship between α‐synuclein levels and GCase activity by analyzing the baseline GCase activity in our *SNCA/SNCA* mice compared to that in wild‐type mice. Significant reductions in GCase activity were observed in the brainstem, midbrain, cortex, and striatum of *SNCA/SNCA* mice. This finding further supports existing data, which demonstrate that increased α‐synuclein is associated with decrease in GCase activity in PD brains and in the SH‐SY5Y cell lines overexpressing SNCA.^15^ Analysis of α‐synuclein protein levels in different brain regions of *SNCA/SNCA* mice demonstrated approximately 50% increase in α‐synuclein levels compared to wild‐type mice in the brainstem, cortex, and striatum. The observed increase was comparable to that previously reported, where a 1‐ to 1.5‐fold increase in α‐synuclein protein expression was observed, when compared to wild‐type endogenous mouse α‐synuclein levels.^31^ We also observed a significant increase in S129 phosphorylation of α‐synuclein in the brainstem, cortex, and striatum of our *SNCA/SNCA* mice compared to that in wild‐type mice. There is increasing evidence that phosphorylation of α‐synuclein may play a pivotal role in α‐synuclein aggregation and formation of Lewy bodies and neurites. Numerous studies reported excessive accumulation of α‐synuclein phosphorylated at residue S129 in the brain of PD patients, where phosphorylated α‐synuclein accounts for up to 90% of total α‐synuclein found within Lewy bodies.^6^, ^32^ Also, phosphorylation of α‐synuclein at residue S129 seems to be aberrantly accumulated in the brain of animal models of synucleinopathies.^32^

We observed a significant increase of GCase activity in the brainstem, midbrain, and cortex following 4mM ambroxol administration in *SNCA/SNCA* mice and approximately 20% reduction of α‐synuclein protein levels in these regions. This further confirms the existence of a reciprocal relationship between α‐synuclein and GCase levels, because the increase in GCase activity in our ambroxol‐treated *SNCA/SNCA* mice led to decrease in α‐synuclein protein levels. Finally, we investigated whether ambroxol was capable of lowering the levels of α‐synuclein phosphorylated at residue S129 and observed more than a 40% reduction in ambroxol‐treated *SNCA/SNCA* mice. This finding is particularly exciting in light of the growing recognition of the importance that phosphorylation of α‐synuclein plays in the pathogenesis of synucleinopathies.^32^ To our knowledge, this is the only study conducted to date that investigates the effect of ambroxol treatment on GCase activity and α‐synuclein in transgenic mice overexpressing human α‐synuclein in the absence of mouse α‐synuclein. Altogether, the ability of ambroxol to decrease both α‐synuclein and S129‐phosphorylated α‐synuclein protein levels is very promising for its future application as a potential drug for treatment of PD and other synucleinopathies, including dementia with Lewy bodies.

No significant changes in HEXB activity were observed in the brainstem, midbrain, cortex, or striatum of wild‐type, *L444P*/+, and *SNCA/SNCA* mice following 4mM ambroxol treatment, suggesting that ambroxol has no effect on lysosomal content. This observation is in contrast to the data obtained from human PD‐*GBA1* fibroblasts, which showed a significant decrease in HEXB activity after ambroxol treatment.^23^ This clearly indicates that further work is required to determine the influence of ambroxol on HEXB.

Collectively, our data show that oral ambroxol is able to increase brain GCase activity in vivo in both wild‐type and transgenic mice. Its chaperone activity appears to be important for targeting GCase for transport to the lysosome. Ambroxol's ability to penetrate the brain–blood barrier, elevate GCase, and reduce α‐synuclein and S129‐phosphorylated α‐synuclein protein levels suggests its potential for development as a treatment for patients with PD and other synucleinopathies.^33^, ^34^, ^35^

## Author Contributions

Study concept and design: A.M.‐R., E.B., A.H.V.S.; data acquisition and analysis: A.M.‐R., L.D., A.H.V.S.; manuscript drafting: A.M.‐R.; manuscript editing: A.M.‐R.; E.B., A.H.V.S.; manuscript final approval: all authors.

## Potential Conflicts of Interest

Nothing to report.
